# A Review of Gene–Property Mapping of Cementitious Materials from the Perspective of Material Genome Approach

**DOI:** 10.3390/ma17153640

**Published:** 2024-07-23

**Authors:** Fei Li, Yan Zhong

**Affiliations:** School of Civil and Resource Engineering, University of Science and Technology Beijing, Beijing 100083, China; zh_ong_yan@163.com

**Keywords:** cementitious material, characteristic genes, properties, mapping relationships

## Abstract

As an important gelling material, cementitious materials are widely used in civil engineering construction. Currently, research on these materials is conducted using experimental and numerical image processing methods, which enable the observation and analysis of structural changes and mechanical properties. These methods are instrumental in designing cementitious materials with specific performance criteria, despite their resource-intensive nature. The material genome approach represents a novel trend in material research and development. The establishment of a material gene database facilitates the rapid and precise determination of relationships between characteristic genes and performance, enabling the bidirectional design of cementitious materials’ composition and properties. This paper reviews the characteristic genes of cementitious materials from nano-, micro-, and macro-scale perspectives. It summarizes the characteristic genes, analyzes expression parameters at various scales, and concludes regarding their relationship to mechanical properties. On the nanoscale, calcium hydrated silicate (C-S-H) is identified as the most important characteristic gene, with the calcium–silicon ratio being the key parameter describing its structure. On the microscale, the pore structure and bubble system are key characteristics, with parameters such as porosity, pore size distribution, pore shape, air content, and the bubble spacing coefficient directly affecting properties like frost resistance, permeability, and compressive strength. On the macroscale, the aggregate emerges as the most important component of cementitious materials. Its shape, angularity, surface texture (grain), crushing index, and water absorption are the main characteristics influencing properties such as chloride ion penetration resistance, viscosity, fluidity, and strength. By analyzing and mapping the relationship between these genes and properties across different scales, this paper offers new insights and establishes a reference framework for the targeted design of cementitious material properties.

## 1. Introduction

The ‘Materials Genome Initiative’ (MGI) is a pivotal strategy for the next generation of transportation infrastructure and is currently a focal point in global competition within the research and development of transportation infrastructure materials [1,2]. In the realm of molecular biology, a gene is a set of information that pertains to its encoding. By analogy, in materials science, genes are viewed as the fundamental units that dictate the macroscopic and microscopic properties of materials [3,4]. The advancement of a material gene database, therefore, enables the prediction of material compositions essential for specific properties and facilitates a reciprocal approach to the design of material ‘gene–property’ relationships.

Cementitious materials, which are multi-phase and non-homogeneous, are primarily based on silicate cement and exhibit a dispersed porous structure [5]. They are characterized by superior compactness, durability, and high strength compared to traditional cement mortars. These attributes render cementitious materials indispensable in various construction fields, including building, road, and water conservancy projects [6]. Current research into the properties of cementitious materials predominantly employs experimental and numerical image processing techniques [7,8,9]. These methodologies facilitate the observation of nano- and microscopic material changes and the subsequent analysis of cementitious material performance. However, the high demand for human and financial resources in these methods cannot be overlooked. Additionally, the validity of previous experimental data is often compromised due to the geographical diversity of data sources, leading to inconsistencies in control variables and consequently, the invalidation of a significant amount of data. This issue impedes the full utilization of experimental data. In contrast, the advent of big data-driven material genome-based strategies marks a paradigm shift in material development [10]. The establishment of a material gene database, integrated with high-throughput computational and machine learning techniques, promises a rapid and precise correlation between material genes and their performance. This approach not only minimizes the production costs of experimental materials but also accelerates the material research and development process, thereby broadening the applicability of cementitious materials.

**Figure 1:**
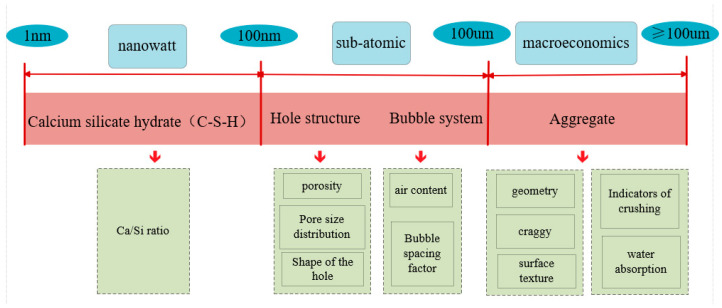
Expression of major characteristic genes corresponding to different scales.

A multiscale approach is an effective strategy for studying non-homogeneous materials. Cementitious materials demonstrate a spectrum of gene expressions across different scales. The interplay and mutual influence of various characteristic genes result in the manifestation of distinct macroscopic properties in cementitious materials [11]. This paper provides a comprehensive summary of the representative characteristic genes of cementitious materials, spanning from the nanoscopic to the macroscopic scales. The principal characteristic gene expressions at each scale are illustrated in Figure 1. At the nanoscopic scale, calcium silicate hydrate (C-S-H) stands out as the predominant characteristic gene. Within the microscopic scale, the focus is on the pore structure and the bubble system. On the macroscopic scale, the aggregate is recognized as the key characteristic gene. The relationships between these main characteristic genes and their respective properties have been delineated, thereby establishing a foundation for the development of a gene database platform.

## 2. Main Characteristic Genes of Cementitious Materials

### 2.1. Nano View: Hydrated Calcium Silicate

**Figure 2:**
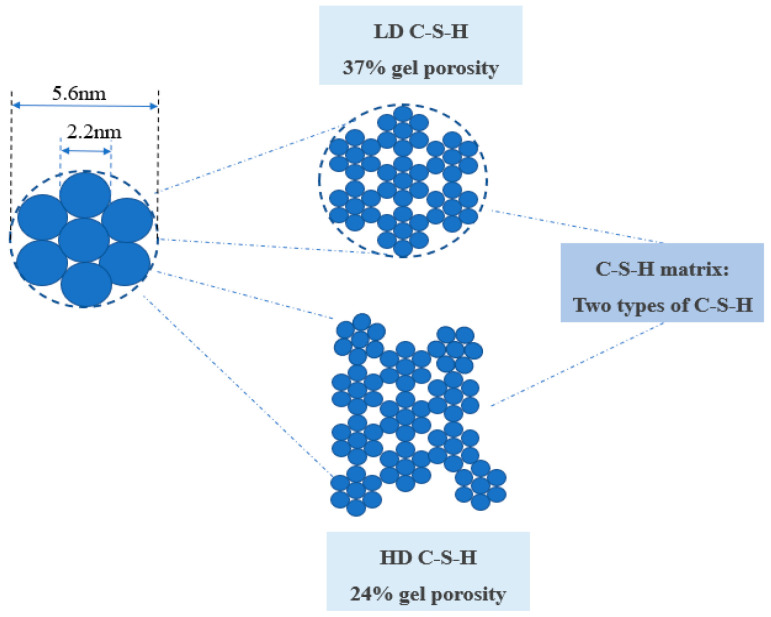
Structure of C-S-H compositions of two different densities.

**Figure 3:**
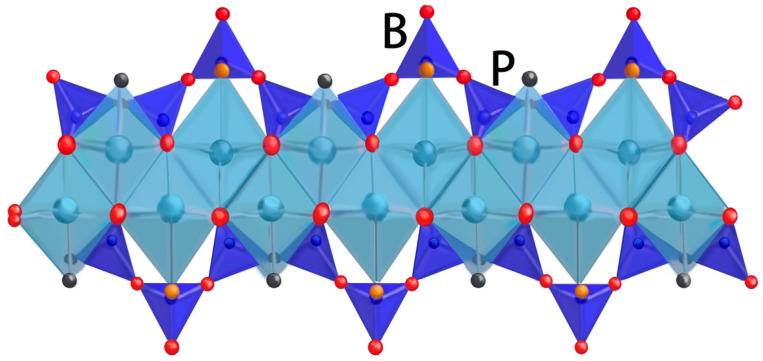
Schematic structure of calcium–silica layer.

**Figure 4:**
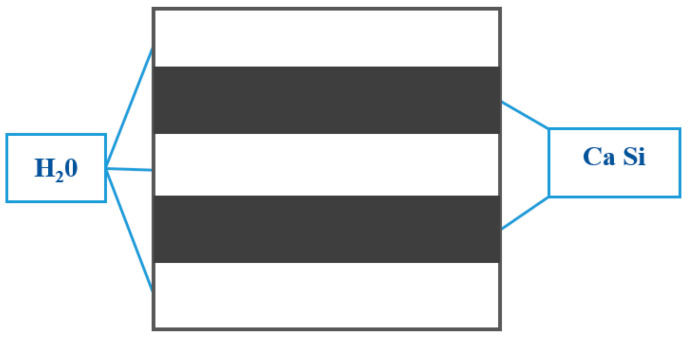
Simple diagram of the side of hydrated calcium silicate.

Constructing a cementitious material system involves the formation of four primary hydration products. The system includes hydrated calcium silicate (C-S-H), calcium hydroxide (CH), ettringite (Aft), and monosulfate (AFm). Hydrated calcium silicate (C-S-H) predominates, constituting approximately 70% of the total content, with calcium hydroxide (CH) comprising an additional 15% to 25% of the total content [12,13]. Consequently, as the paramount hydration product in cementitious materials, C-S-H dictates the strength, deformation, and service life of these materials. It also represents the most critical nanoscopic characteristic gene within the material matrix. The hardened cement paste encompasses two distinct types of C-S-H, differentiated by their structural attributes and packing densities. The first is a low-density, high-strength C-S-H with 37% porosity, known as the external hydration end product (OP). The second is a high-density, low-volume-ratio C-S-H with 24% porosity, designated as the internal hydration end product (IP). Both types of hydration products are composed of nano-scale spherical particles. The spherical particles that constitute the cementitious material have an average diameter of approximately 5.6 nm. The fundamental particles within these spheres, which dictate their structure, measure about 2.2 nm in diameter and have a density of approximately 2.8 g/cm^3^ [14]. The external product (OP) develops in a scaly pattern, radiating outward from the cement clinker, forming clusters along the vertical axis and presenting a more extensive external pore structure than the internal product (IP). In contrast, IP is characterized by a uniform and compact arrangement of spherical particles, 4–6 nm in diameter, which form tight sheets, circular, or elliptical configurations with external pore diameters under 10 nm, as illustrated in Figure 2. Utilizing molecular dynamics simulations, Roland J.-M. Pellenq [15] has delineated the atomic structure of C-S-H, known as the Tobermorite model, resembling the structure of C-S-H gel. This model is composed of a series of stacked calcium–silica layers, with calcium ions and water molecules interspersed between each layer. As shown in Figure 3, each calcium–silica layer contains a central calcium–oxygen octahedral layer, with silica–oxygen tetrahedral chains extending from both the top and bottom. These layers are positioned to completely overlap, interconnected by Si-O-Si chemical bonds. Merlino [15] suggests that after a vertical translation, the layers align perfectly, reinforced by these bonds. Hamid [16], however, posits that these neighboring layers remain somewhat independent, with minimal chemical bonding and a slight horizontal offset. Expanding on this, Xiaolan Ma [17] envisions C-S-H as a layered structure of three to four compact cylinders, as depicted in Figure 4. The interlayer spacing within C-S-H has been measured at 0.98 ± 0.01 nm. The Tobermorite model offers a more nuanced understanding of the C-S-H gel’s atomic structure, clarifying its lamellar configuration and the dynamics of its formation and change, thereby providing a robust theoretical foundation for further studies on the atomic structure of C-S-H gels.

**Figure 5:**
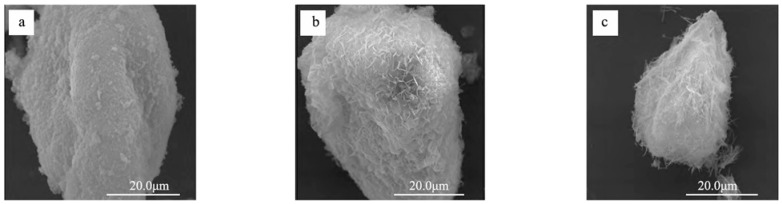
Schematic representation of the morphology of hydrated calcium silicate at different Ca/Si ratios [22]. (**a**) C-S-H is agglomerated, granular, and flaky at 1.5 Ca/Si ratio, (**b**) C-S-H is in the form of short and thick acicular rods and longer network fibers at 1.5–2.0 Ca/Si ratio, (**c**) C-S-H is in the form of long and thin acicular rods and longer network fibers at 1.5–2.0 Ca/Si ratio.

The calcium–silicon ratio is the most important parameter describing the structure of hydrated calcium silicate. The calcium–silicon ratio of hydrated calcium silicate gels generally fluctuates around 1.5. The calcium–silicon ratio of the external product (OP) is greater than 1.5, whereas that of the internal product (IP) is less than 1.5 [18,19,20,21]. As the calcium–silicon ratio increases, the morphology of hydrated calcium silicate changes [22] (Figure 5 below). When the calcium–silicon ratio is less than 1.5, most of the hydrated calcium silicate aggregates in the form of surface-dense clusters, granules, and flakes. As the calcium–silicon ratio increases from 1.5 to 2.0, calcium silicate hydrate transitions to a short, thick needle-and-rod structure or a longer reticulated fibrous structure. When the calcium–silicon ratio increases beyond 2.0, the hydrated calcium silicate is primarily characterized by elongated needle-and-rod structures or long, loose reticulated fibrous structures [23,24,25,26] (Table 1 below).

### 2.2. Microscopic: Pore Structure and Bubble Systems

#### 2.2.1. Hole Structure

**Figure 6:**
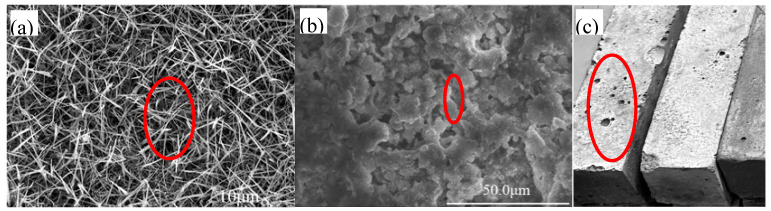
Schematic representation of capillary pores, gel pores, and stomata in cementitious materials [31,32]: (**a**) capillary pores, (**b**) gel pores, (**c**) stomata.

In cementitious materials, pores are classified according to their impact on concrete’s strength, quality, and properties into categories: harmless pores (less than 20 nm), less harmful pores (20 to 50 nm), harmful pores (50 to 200 nm), and more harmful pores (greater than 200 nm). Enhancing the mechanical properties of cementitious materials involves increasing the prevalence of pores smaller than 50 nm while decreasing those larger than 100 nm [27]. The classification of pores by size includes capillary pores, gel pores, and air pores [28,29,30], as depicted in Figure 6. Capillary pores, which are unoccupied spaces during the hydration process, form continuous network-like structures that range from 10 nm to 10 µm. Gel pores, known as interlayer pores within hydrated calcium silicate, make up 28% of the hydrated calcium silicate gel’s volume, with sizes between 1.5 and 2.0 nm, matching the dimensions of a water molecule. Pore formation is partly attributed to the air introduced during the material’s preparation, which persists as air bubbles within the mixture. Furthermore, the incorporation of water-reducing or air-entraining agents during mixing encapsulates air, forming bubbles with diameters generally exceeding 10 µm and presenting a spherical shape [31,32], as illustrated in Figure 6.

#### 2.2.2. Bubble System

The bubble system is a pivotal characteristic in understanding the genetic makeup of cementitious materials. Researchers primarily assess this system using two key metrics: the air content and the bubble spacing coefficient [33,34,35,36,37,38]. In cementitious materials with low air content, the microstructure is primarily composed of smaller pores such as gel pores, transition pores, and capillary pores. An increase in gas content results in a proportional rise in the prevalence of larger pores, which in turn increases the overall porosity. During the hydration process, the small bubbles within the material coalesce to form larger bubbles. This coalescence, along with the enlargement of bubble spacing, leads to a continuous decline in the structural integrity of the cementitious material.

Li Jianxin [39] noted that an increased air content in cement mortar corresponds to higher porosity and a greater total pore volume under constant proportions, with these measures gradually escalating. The proportion of pores larger than 50 nm also increases. Specifically, when the air content rose from 2.3% to 6.2%, the pore spacing was observed to contract from 0.199 mm to 0.072 mm, indicating that the air content in cement mortar can significantly refine the pore structure parameters.

Xu Wenying [40] discovered, through the application of the fast freezing method and slump test research, that the bubble spacing coefficient in cementitious materials first diminishes and then ascends with the escalation of air content. At a gas content of 5.4%, the bubble spacing coefficient reached a nadir of 146.682 µm. Further increments in gas content led to an increase in the bubble spacing coefficient. This upsurge is attributed to the aggregation of numerous minute bubbles into larger ones, consequently amplifying the inter-bubble spacing coefficient.

Chen Ziyu [41] employed nuclear magnetic resonance (NMR) and mercury intrusion porosimetry (MIP) to examine low-temperature-cured cementitious materials, uncovering that the porosity within these materials, particularly for those 35 days old, progressively increased alongside the elevation in gas content. Notably, there was a heightened frequency of transition pores, macropores, and gel pores.

Aggregates are the preeminent constituents of cementitious materials, constituting 60% to 80% of the material composition. Notably, coarse aggregates provide a structural framework within cementitious materials [42,43,44,45,46,47,48]. The expression of characteristic genes in aggregates can be categorized into three dimensions: shape properties, encompassing form, angularity, and surface texture (grain); strength properties, focusing predominantly on the crushing index; and physical properties, with a primary emphasis on water absorption [49,50,51,52].

### 2.3. Macro: Aggregates

#### 2.3.1. Aggregate Shape

Characterizing the shape of particles is crucial in various scientific disciplines, and the primary parameters include perimeter, area, and the lengths of the short and long axes, along with contour shape [53]. In this investigation, we focus extensively on contour shape parameters, which are instrumental in describing the overall morphology of particles. Specifically, we use terms such as ‘spherical’, ‘needle’, and ‘flake’ to classify their shapes. The concept of sphericity, introduced by Wadll [54], is pivotal in quantifying the degree to which particle shapes deviate from a spherical form, as illustrated in Equation (1). A sphericity value closer to 1 suggests a more spherical three-dimensional shape of the aggregate particles [55,56]. Additionally, the needle coefficient, which is the ratio of the long axis to the short axis of the particle’s equivalent ellipse, indicates the acicular nature of the particles; a higher ratio implies a more needle-like shape. Conversely, the flake coefficient, the ratio of the particle’s center axis to its short axis, reveals the flaky nature of the particles, with a lower ratio indicating a more pronounced flakiness.

(1) $$s=\sqrt[{3}]{36\pi v_{A}^{2}}∕s_{A}$$

where s is the particle sphericity, $v_{A}$ is the volume of the coarse aggregate, and $s_{A}$ is the surface area of the coarse aggregate.

#### 2.3.2. Aggregate Angles

**Figure 7:**
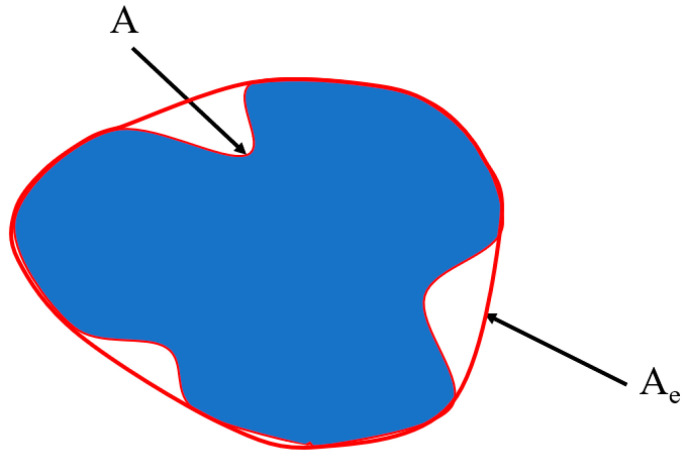
Convex polygon method schematic.

The angularity of aggregate particles is a pivotal measure of their sharpness at the corners, reflecting both the concavity of the contour and the convexity of the corners. A lower angularity is indicative of a smoother particle surface and enhanced convexity, which are significant in the context of material properties [57,58,59,60]. The angularity coefficient, a standard metric calculated via the convex polygon method, is depicted in Figure 7 and serves as a reliable indicator of aggregate angularity. Within the realm of cementitious materials, the compressive strength exhibits a modest increase at lower values of the coarse aggregate angularity coefficient. In contrast, a substantial decrease in compressive strength is observed when the coarse aggregate angularity coefficient surpasses the threshold of 1.5 [61].

(2) $$AI\mathrm{e}=\frac{A_{e}}{A}$$

where AIⅇ is the convex polygon normal angularity index, $A_{e}$ is the area of the convex polygon, A is the area of the coarse aggregate profile.

#### 2.3.3. Surface Texture

Surface texture is a critical parameter for assessing the concavity and convexity of aggregate particle surfaces, offering a precise reflection of their textural characteristics [62,63,64,65]. AIMS II, the Aggregate Image Measurement System utilized in the United States, is an advanced tool for swiftly capturing aggregate profile attributes. It provides a comprehensive characterization of particle angularity, composition, and surface texture. This system categorizes surface texture into four distinct classes, each defined by specific angularity indicators. These indicators span a scale from 0 to 10,000, where a lower value denotes a smoother aggregate particle surface. The lowest possible value, 0, signifies an ideal, perfectly smooth particle surface texture. The detailed classifications, as delineated in Table 2, are as follows.

#### 2.3.4. Crushing Indicators

The crushing index is a critical metric for gauging an aggregate’s resilience to external compressive forces, quantified by the mass percentage of fines—aggregate particles smaller than a defined size—resulting from a standardized crushing test, as exemplified by Equation (3). This index provides an indirect yet valuable reflection of the intrinsic strength properties of the material.

(3) $$\delta_{a}=\frac{m_{0}-m_{1}}{m_{0}}*100\%$$

where $\delta_{a}$ is the crushing index of the aggregate, $m_{0}$ is the mass of the specimen, $m_{1}$ is the mass of the specimen sieved after the crushing test.

In accordance with the national standard GB/T 14685-2022 [66], construction materials are meticulously classified into distinct categories: Class I, Class II, and Class III, predicated on stringent technical specifications. Class I, reserved for high-performance concrete applications, is specified for use in concrete mixtures with strength grades surpassing C60. For moderate-strength concrete, Class II is the designated category, accommodating strength grades between C30 and C60. Conversely, Class III is earmarked for use in concrete with strength grades that fall below the C30 threshold. A comprehensive delineation of these classifications is meticulously presented in Table 3, spanning across the ensuing pages for detailed examination [67,68].

In response to the pressing environmental concerns stemming from the disposal of substantial construction waste, and in alignment with national policies advocating for sustainable environmental development, as well as the imperative to conserve natural resources, the academic community has increasingly directed its focus towards the utilization of recycled aggregates in the field of materials engineering [69,70]. These recycled aggregates are derived from the crushing of construction waste, retaining old cement mortar on their surfaces. When juxtaposed with natural aggregates, recycled aggregates exhibit a tendency towards larger overall particle sizes, coupled with relatively lower apparent and bulk densities. Notably, the crushing index of recycled aggregates commonly surpasses that of their natural counterparts, with values estimated to be three to four times higher, specifically within the range of 14.2 to 23.1, as detailed in Table 4 [69,70]. This disparity can be primarily attributed to two fundamental factors [71,72,73,74,75,76]:

1. The encapsulation of secondary aggregates within cement mortar, which, due to its relatively lower strength, yields a higher crushing index for recycled aggregates.
2. The formation of cracks within the stone during the crushing process, which diminishes the strength of the recycled aggregate. Table 4 compiles a comprehensive overview of the crushing index data for both natural and recycled aggregates, as documented by a multitude of scholars.

#### 2.3.5. Water Absorption

In the field of material science, the water absorption of aggregate is a pivotal property, signifying the extent to which an aggregate can absorb water as it transitions from a dried state to a state of saturation at the surface under standard atmospheric conditions, and is conventionally quantified by a percentage (as illustrated by Equation (4)).

(4) $$\omega_{x}=\frac{m_{f}-m_{a}}{m_{a}}*100\%$$

where $\omega_{x}$ is the water absorption of the aggregate, $m_{f}$ is the saturated surface dry mass of the aggregate, $m_{a}$ is the dried mass.

The national standard GB/T 14685-2022 [66], entitled ‘Pebbles and Crushed Stone for Construction’, meticulously classifies the water absorption of aggregates into three distinct tiers predicated on stringent technical criteria. For an exhaustive delineation of these classifications, the reader is directed to Table 5, which follows.

The water absorption characteristic of recycled aggregates typically exceeds that observed in natural aggregates, with values typically ranging from 4 to 10 percent, which is notably 3 to 5 times greater than that of natural aggregates. This disparity arises primarily due to two factors [81,82,83].

1. The process of stone crushing generates cracks both on the surface and within the natural aggregate, facilitating the penetration of cement mortar into these fissures, thereby augmenting the water absorption and rate of the aggregate.
2. The reduction in particle size due to stone crushing increases the specific surface area of recycled aggregates, enhancing their contact area with cement mortar and, in turn, their water adsorption capacity.

The correlation between water absorption and the crushing index is robust, exhibiting a positive proportional relationship with correlation coefficients consistently above 0.94. This strong correlation supports the viability of employing the water absorption rate as an indicator for assessing the crushing index of aggregates in practical engineering applications. Table 6 encapsulates the synthesis of findings from pertinent scholarly research on the relationship between the water absorption rate and the crushing index of aggregates, where x represents the crushing index and y denotes water absorption.

## 3. Relationship between the Main Characteristic Genes of Cementitious Materials and Performance Mapping

### 3.1. Mapping of Hydrated Calcium Silicate to Properties

**Figure 8:**
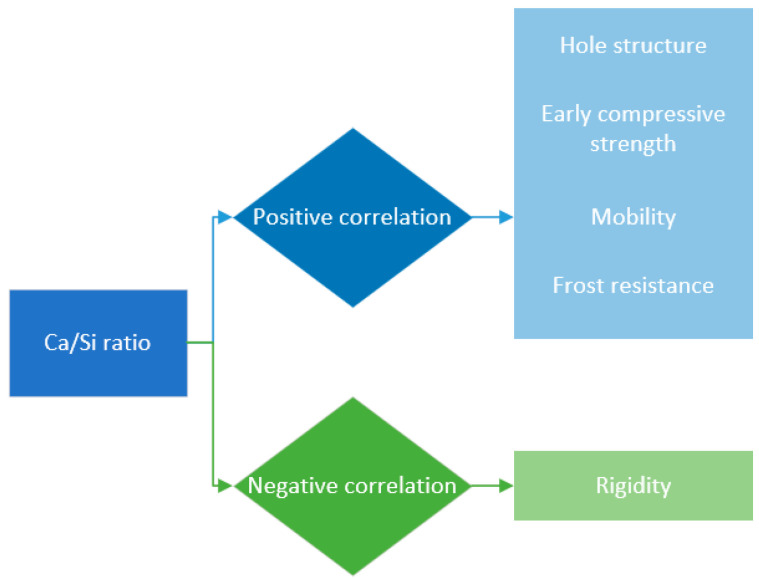
Positive and negative correlation between calcium–silicon ratio and performance.

The calcium–silica ratio, a crucial determinant in the microstructure of hydrated calcium silicate, exerts a profound influence on the mechanical properties of cementitious materials. An increment in this ratio is correlated with a gradual reduction in the density of hydrated calcium silicate and a concomitant decline in the stability of its lamellar structure, thereby diminishing the material’s capacity to withstand external deformation. The evolution of Young’s modulus and the modulus of elasticity follows a biphasic trend, initially ascending then descending. This behavior is concomitant with alterations in the pore structure of cementitious materials, which paradoxically enhance early compressive strength but concurrently impair flow properties and frost resistance. Additionally, there is an initial surge followed by a decline in material stiffness [87,88,89,90], as illustrated in Figure 8. At a calcium–silica ratio of 1.1, the density of hydrated calcium silicate is recorded at 2.57 g/cm^3^, with an average silica chain length of 7.37 nanometers. Upon elevating the ratio to 1.9, the density plummets to 2.34 g/cm^3^, and the average silica chain length contracts to 2.27 nanometers, reflecting a density decrease of 8.9% and a chain length reduction of 5.10%. The ensuing loosening of the cementitious material’s pore structure precipitates a reduction in early compressive strength. At calcium–silica ratios of 1.2 and 1.5, the flow of cementitious materials is quantified at 165 mm and 158 mm, respectively, signifying a decrement of 7 mm. The accrual in compressive strength begins to plateau, achieving increments of 12.2% and 10.9%, respectively. Following cycles of freezing and thawing, the material incurs strength losses at rates of 5.7% and 6.4%, respectively. The calcium–silica ratio of 1.7 marks the zenith for early strength of cementitious materials, concurrently yielding peak values for Young’s modulus and elastic modulus, which are 64.8 GPa and 70.1 GPa, respectively. Beyond this ratio, an increment from 1.7 to 2.1 induces a downward trajectory in the elastic modulus, signifying a decrement in the stiffness of cementitious materials from 27 GPa to 20 GPa [91,92,93,94,95,96].

### 3.2. Pore Structure and Performance Mapping Relationship

Porosity, pore size distribution, and pore shape are pivotal parameters in characterizing the pore structure of cementitious materials [97,98,99,100,101]. Recent scholarly work has underscored the direct influence of pore structure on key properties such as the frost resistance, permeability, and compressive strength of these materials.

Yan Xiluo [102] has notably posited that cementitious materials with harmless or minimally harmful pore structures can effectively isolate capillary pores, thereby enhancing both the frost resistance and bond strength. Furthermore, Academician Wu Zhongwei of the Chinese Academy of Sciences [27] has elucidated that pore structure is instrumental in facilitating access to groundwater and sustaining the hydration of cementitious mixes, which in turn provides a conducive environment for the growth of the material and allows for the adjustment of the pore’s basic structure to achieve optimal quality and properties. It has been observed that an increase in the number of pores less than 50 nm and a reduction in those larger than 100 nm significantly bolster the frost resistance of concrete.

In terms of permeability, Powers [30] pioneered the correlation between the permeability coefficient and the porosity of concrete, highlighting that permeability escalates with rising porosity, particularly surpassing the 25% threshold. Wu Zhongwei also emphasized that a well-graded pore structure can substantially improve permeability.

Jiang Linhua [103] has investigated the link between pore structure and the strength of cementitious composites (as illustrated by Equations (5) and (6)), identifying a robust linear correlation between total porosity and compressive as well as flexural strength.

(5) $$f_{f}=10.29-0.20P$$

(6) $$f_{c}=127.04-2.93P$$

where $f_{f}$ is the flexural strength, $f_{c}$ is the compressive strength, P is the total porosity.

The empirical regression equation encapsulates the relationship between pore size distribution and the flexural strength of cementitious materials, providing a quantitative framework for understanding the impact of microstructural characteristics on mechanical performance (as illustrated by Equation (7)).

(7) $$f_{f}=11.60-0.08P_{<20}-0.36P_{20\sim 50}-0.21P_{50\sim 100}-0.45P_{>100}$$

The regression analysis elucidates the nuanced influence of pore size distribution on the mechanical properties of cementitious materials, specifically compressive and flexural strength. Notably, pores measuring less than 20 nm minimally affect compressive strength, while those exceeding 100 nm significantly impair it. Pore sizes between 20 nm and 50 nm are more detrimental to compressive strength than those ranging from 50 to 100 nm. Building on this regression equation analysis, Jiang Linhua [103] employed finite element methods for computational simulations, yielding three distinct models: a model with a central square pore, one with four symmetrical circular pores, and another with four symmetrical square pores. These simulations revealed that at a constant porosity, smaller pores are associated with higher compressive strength. Among the models, the one featuring circular pores demonstrated superior strength over its square pore counterpart. Optimal pore structure, marked by low porosity, appropriately sized and graded small pores, and a prevalence of rounded pores, is crucial for materials with high strength and enduring durability.

### 3.3. Bubble System and Performance Mapping Relationship

Uniformly distributed, stable, and sealed air bubbles in cementitious materials significantly diminish inter-aggregate collision and effectively sequester water spillage within the capillary channels. Simultaneously, they serve the dual function of ‘ball bearings’, enhancing the cement paste’s lubricity, which in turn bolsters the materials’ durability and compressive strength, and is pivotal for their frost resistance [104,105,106,107,108].

The ideal pore structure and properties of these materials are typically attained with an air content ranging from 3% to 5% [109,110]. Overabundance of air results in excessive porosity, manifesting as a honeycomb structure that sacrifices compactness and leads to an initial surge in yield stress followed by a decline. Conversely, insufficient air content compromises compatibility and frost resistance, and predisposes materials to cracking.

The air bubble system plays a crucial role in determining the frost resistance of cementitious materials. Xie Jian [111] explored the correlation between air-entraining agents and the frost resistance of these materials, focusing on their pore structure. The study revealed that the addition of air-entraining agents facilitates the creation of a certain proportion of harmless pores within the mixture, while also significantly reducing the presence of multi-hazardous pores. This leads to an improvement in frost resistance, as evidenced by an increase in pore diameter and porosity following freeze–thaw cycles. However, this enhancement in frost resistance is accompanied by a decrease in mechanical properties. Li Jianxin [39] proposed that the frost durability of cement mortar is positively correlated with air content, though it is constrained by the material’s strength. Liu Pengfei [112] conducted indoor tests on cementitious materials with different air contents to evaluate the effect of bubble characteristics on durability. The results indicated that hardened cementitious materials with a bubble spacing coefficient below 300 μm and an average chord length of bubbles below 200 μm demonstrate enhanced resistance to frost and salt freezing. Liu Xinchao [108] performed freeze–thaw cycle tests on cementitious materials with varying air contents. The findings suggest that as the air content rises to 4.3%, the frost resistance of the materials increases, with the mixture achieving optimal frost resistance and mechanical properties at this threshold. Beyond this point, an increase in air content is detrimental to the frost resistance of cementitious materials. These insights are essential for the study of mechanical properties in cement concrete pavements.

The air bubble system exerts a significant influence on the strength properties of cementitious materials. Wenying Xu [40] meticulously scrutinized the impact of air content and bubble characteristics on these properties. The findings indicate that a judicious increase in the air content can markedly enhance the compressive strength and frost resistance of cementitious materials. At an air content of 5.4%, the hardened material exhibits a bubble spacing coefficient of 146.682 μm and an average bubble diameter of 71.948 μm, which culminate in exceptional compressive strength and frost resistance. This is exemplified by a 28-day strength of up to 49.9 MPa, an F300 mass loss rate of 0.16%, and a relative elastic modulus of 94%. Recently, Xu Cundong [107] utilized NMR technology to delineate the correlation between the fractal dimension of pore volume and the compressive strength of cementitious materials. The study deeply analyzed the model fitting the relationship between compressive strength and the maximum fractal dimension ($D_{max}$) during freeze–thaw cycles, as detailed in Table 7. It revealed that elevating air content can notably refine the internal pore structure, consequently bolstering the mechanical properties, including compressive strength, of materials when exposed to saline frost conditions.

### 3.4. Aggregate–Property Mapping Relationships

#### 3.4.1. Aggregate Shape

Aggregate shape significantly influences the pore structure of cementitious materials, thereby affecting their resistance to chloride ion penetration, as well as their viscosity and fluidity.

An increased proportion of flattened or elongated aggregates leads to greater overlap and a larger contact area among aggregate layers. This results in a reduction in the mix’s porosity, which in turn decreases the likelihood of chloride ions penetrating the flaky aggregates. Consequently, the chloride ion permeability coefficient of cementitious materials is reduced with an increased dosage of lamellar coarse aggregates. A threshold is observed; when the flake content equals 15%, the permeability of cementitious materials to chloride ions reaches its nadir [113,114]. Furthermore, cementitious materials that incorporate aggregates with a low percentage of acicular flakes demonstrate reduced viscosity and enhanced fluidity [115].

#### 3.4.2. Aggregate Angles

Aggregate angularity significantly influences the flow, viscosity, strength, and peak shear stress of cementitious materials. As the angularity diminishes, the viscosity of the mix decreases, which in turn enhances the flow deformation capacity [116,117,118,119]. Xueqin Zhang [48] investigated the quantitative relationship between aggregate characterization parameters and the properties of cementitious materials. The findings indicate that increased aggregate angularity is associated with greater resistance and reduced fluidity during the mixing and pouring processes. Bu Yin [120], through experimental analysis, observed that reduced aggregate angularity correlates with decreased peak shear force and a significant reduction in peak shear stress, suggesting that lower angularity improves the ease of mix construction. Liang Ruheng [121] employed the angularity coefficient to assess the shape of coarse aggregate grains, noting that values exceeding 1.5 are linked to a substantial decline in the workability and mechanical properties of cementitious materials. Shapes that are spherical or nearly spherical are more beneficial to the performance of cementitious materials, whereas angular shapes are deemed less favorable.

#### 3.4.3. Surface Texture

Surface texture is a pivotal factor affecting the slip resistance of cementitious materials. Zhang Kaiyin [122] conducted a molecular-level investigation using molecular dynamics simulation software. The findings indicated that a decrease in porosity leads to a reduction in the surface texture’s effectiveness, thereby diminishing the slip resistance of the materials. It has been observed that a rougher aggregate surface texture enriches the material’s texture, subsequently enhancing both durability and anti-slip performance. To date, scant research has addressed the relationship between the surface texture of aggregates and the performance of cementitious materials; instead, the emphasis has been on how coarse aggregate morphology affects the performance of asphalt mixtures. Chen Guoming and Tan Yiqiu [123] used a laser profiler for precise measurement of aggregate surface texture, employing the arithmetic mean deviation (Ra) as a quantification metric. Their study demonstrated a positive correlation between an increased Ra value of coarse aggregates and improved low-temperature crack resistance. Furthermore, Zhou Chunxiu and Chen Guoming [124] explored the fractal characteristics of coarse aggregate surface textures and noted that increased surface roughness progressively strengthens the mixture’s resistance to low-temperature cracking.

#### 3.4.4. Crushing Indicators

The crushing index of aggregates exerts a significant influence on key properties of cementitious materials, including compressive strength, flexural strength, modulus of elasticity, and permeability coefficient. Shen Yanqiu [125] investigated these properties in relation to cementitious materials with crushing indices ranging from 3.5% to 9.5%. The findings indicated a negative correlation between the crushing index and both compressive and flexural strengths. In another study, Ma Minchao and Zhou Yaoxu [126] assessed cementitious materials of C30, C40, and C50 grades with crushing indices of 25.1%, 22.6%, and 13.2%, respectively. Their research concluded that for C30-grade materials, the aggregate’s crushing index is not the predominant factor affecting strength. In contrast, for C40 and C50 grades, the crushing index was identified as the primary factor restricting strength due to the failure at the aggregate’s weak points during the 28-day compression tests. Additionally, it was observed that the modulus of elasticity increases as the crushing index decreases. For instance, the modulus of elasticity of material with 13.2% crushing index is 2.23 × 10^4^ GPa higher than that of material with 25.1% crushing index, and 1.79 × 10^4^ GPa higher than that of cementitious material with 22.6% crushing index. The scholars Liu Dai and Dong Changzhen [127] analyzed the relationship between cementitious materials with 11.8% and 4.9% crushing index and their elastic modulus. It was found that the compressive strength of the material with high crushing index decreases by 2.6 MPa, and the elastic modulus decreases by 0.4 × 10^10^ MPa. The crushing index adversely affects the deformation resistance of cementitious materials. Scholars Wang Changyin and Ge Folding Sheng [113] studied the effect of crushed aggregate on the impermeability of concrete. They found that the permeability coefficients of cementitious materials with crushing indexes of 25%, 18%, and 10% were 9.95 × 10^−9^ cm·s^−1^, 5.34 × 10^−9^ cm·s^−1^, and 2.36 × 10^−9^ cm·s^−1^, respectively. It was concluded that there was an exponential relationship between the aggregate crushing index and the permeability coefficient. The permeability coefficient increases with the increase in the value of crushing.

#### 3.4.5. Water Absorption

The water absorption of aggregates is a critical factor that significantly influences the slump, fluidity, drying shrinkage capacity, and compressive strength of cementitious materials. Aggregates typically have a water absorption rate controlled within 1%. When this rate is exceeded, the slump of the material decreases significantly, and its fluidity diminishes over time, which can compromise compliance with on-site construction requirements [128]. In the early stages of cementitious materials’ hardening, higher water absorption by aggregates postpones the onset of drying and shrinkage. However, as time elapses, the water within the aggregates evaporates, causing the pore spaces in the cementitious materials to expand and the drying shrinkage to increase [129]. The compressive strength of these materials is also adversely affected by increased aggregate water absorption. Ravindrarajah’s study [130] reports a reduction in compressive strength ranging from 5% to 24%. Rama Murthy [131] suggests that the reduction in strength can extend from 15% to 42%. Mandal’s research [132], comparing the compressive strength of recycled concrete at different ages, indicates a reduction of approximately 15%. Fan Chunxi and colleagues [130], through their experimental studies, observed a decrease in the strength of cementitious materials by 22% to 31% after a 28-day standard curing period. Wang Dongtao’s study [128], utilizing experimental methods, presents a schematic diagram that correlates the morphology of cementitious materials with different water absorption levels to their compressive strength. Materials with water absorption below 1% are found to have a compact structure with minimal water seepage, yielding a compressive strength of 45.2 MPa. Conversely, materials with water absorption above 1% display larger pores on the surface and significant water seepage, resulting in a compressive strength of 32.2 MPa.

## 4. Summary

In this study, we have characterized the principal genetic features of cementitious materials at the nano-, micro-, and macro-scales. Additionally, we have detailed the interplay between these genetic features and the resultant properties of the cementitious materials.

1. From a nano-scale perspective, hydrated calcium silicate (C-S-H) emerges as the most critical characteristic component of cementitious materials. It is the calcium-to-silicon ratio that serves as the pivotal parameter in describing the structure of C-S-H. This ratio influences the material’s categorization into two distinct forms based on stacking density: OPC, characterized by its low density and substantial porosity of 37%, and IPC, known for its high density and reduced porosity of 24%. As the calcium-to-silicon ratio escalates, the morphological characteristics of C-S-H undergo a transformation, shifting from dense agglomerates to more elongated, needle-like rods, and then to loose, network-like fibrous structures. This transition is accompanied by an increase in early compressive strength, a decrease in fluidity, and a decline in frost resistance.
2. From a microscopic perspective, the pore structure and the bubble system are key characteristics of cementitious materials. These features, including porosity, the distribution of pore sizes, the configuration of pores, the volume of entrained air, and the bubble spacing factor, are critical indicators of the material’s properties. They significantly influence the freeze–thaw resistance, permeability, and compressive strength of cementitious materials.
  ① Porosity: When porosity is below 20%, the permeability of cementitious materials remains minimal. Conversely, when porosity exceeds 25%, permeability notably escalates.
  ② Pore size distribution: The more superior the frost resistance and the greater the compressive strength of cementitious materials, the more numerous the pores under 50 nm and the fewer the pores above 100 nm.
  ③ Shape of holes: Maintaining uniform porosity, cementitious materials with circular pores display enhanced strength relative to those with square pores.
  ④ Air content: Typically, the air content within cementitious materials is confined to a range of 3% to 5%. An air content of approximately 4.3% is associated with the optimal balance of frost resistance and mechanical properties in these materials.
3. From a macroscopic perspective, the aggregate is a defining characteristic of cementitious materials. The properties of the aggregate, such as its shape, angularity, surface texture, crushing strength index, and water absorption capacity, are fundamental in determining the performance of these materials. Notably, these attributes substantially influence the chloride ion penetration resistance, rheological properties, including viscosity and fluidity, as well as the mechanical strength of cementitious materials.
  ① Shape: Aggregates with higher sphericity and cubic shapes are conducive to enhanced performance in cementitious materials.
  ② Angularity: An angularity coefficient above 1.5 adversely affects the mechanical and operational properties of cementitious materials. The performance is optimized with spherical shapes, slightly reduced with near-spherical shapes, and is minimal with angular shapes.
  ③ Surface texture: Low surface texture correlates with reduced slip resistance, while a higher texture enhances durability and anti-slip characteristics of cementitious materials.
  ④ Crushing index: An elevated crushing index is associated with reduced compressive strength and modulus of elasticity. Cementitious materials with crushing indices of 25%, 18%, and 10% demonstrate a gradient in elastic modulus, with the highest index showing a decrease of approximately 2 × 10^4^ GPa compared to the lowest. Correspondingly, an increased permeability coefficient is observed, with the highest index having a coefficient approximately 2.06 × 10^−9^ cm·s^−1^ greater than the lowest.
  ⑤ Water absorption: Typically, the water absorption rate should not exceed 1%. Exceeding this threshold can significantly reduce the slump and fluidity of the material and may result in a compressive strength reduction within the range of 15% to 42%.

The correlation analysis between the characterized properties and their performance across various scales is presented in Table 8 below.

## 5. Research Outlook

The generalization of characteristic properties marks the preliminary phase in the genomic project’s implementation. For cementitious materials, the genomic project’s realization in practical applications hinges on the integration of machine learning algorithms. The construction of a characteristic property database and the development of predictive performance models represent critical dimensions of current research endeavors.

1. The establishment of a standardized, extensive database is essential for the accuracy of performance forecasts. A challenge arises from the utilization of databases compiled from a multitude of previous experimental studies: the inconsistency in test materials and environments, the breadth of data sources, and the suboptimal standardization. Therefore, it is imperative to address the issues of standardization and precision in the creation of subsequent databases for cementitious materials.
2. For performance prediction, machine learning algorithms have become highly sophisticated. Leveraging high-throughput algorithms enables the integration of artificial intelligence with extensive databases, facilitating the establishment of correlations between the characteristic properties and performance of cementitious materials. This approach can identify the optimal material composition and structure to meet specific property requirements in complex cementitious materials. Bidirectional optimization of material properties and their genetic factors is pursued.

While this paper offers insights into specific characteristic properties of cementitious materials, existing research predominantly examines the performance of isolated properties. In practice, characteristic properties exhibit interdependencies rather than being wholly independent. Therefore, subsequent studies should prioritize the development of a comprehensive characteristic property database. Future research should explore the intricate interplay among characteristic properties, construct predictive models that link these properties to the macroscopic behavior of cementitious materials, identify optimal composition ratios, and realize a harmonized bidirectional design process that integrates material properties with their macroscopic manifestations.

## Figures and Tables

**Table 1:** Relationship between calcium–silicon ratio and C-S-H morphological characteristics.

Ca/Si Ratio	C-S-H Morphological Characterization
<1.5	Clusters, granules, flakes
1.5 to 2.0	Short thick needle-like rods, longer network fibers
>2.0	Elongated needle-like rods, loose network fibers

**Table 2:** Classification of aggregate textures.

	Low Texture	Medial Texture	High Grain (Texture)	Extremely High Texture
Range of indicators	0 ≤ X ≤ 2100	2100 ≤ X ≤ 3975	3975 ≤ X ≤ 5400	5400 ≤ X ≤ 10,000

**Table 3:** Aggregate crushing index by technology.

Form		Class I	Class II	Class III
Crushing index/%	crushed or broken rock	≤10	≤20	≤30
	pebbles	≤12	≤14	≤16

**Table 4:** Comparison of crushing index results for natural and recycled aggregates.

Literature Sources	Natural Aggregate Crushing Index/%	Recycled Aggregate Crushing Index/%	Recycled Aggregate/Natural Aggregate
literature [77]	4.04	15.2	3.76
literature [78]	6.20	16.5	2.66
literature [79]	4.85	19.17	3.95
literature [80]	4.80	15.10	3.15

**Table 5:** Aggregate water absorption classified according to technical requirements.

Form	Class I	Class II	Class III
Water absorption/%	≤1.0	≤2.0	≤2.5

**Table 6:** Relationship between aggregate water absorption and crushing index fitting.

Literature Sources	Fit Function	Correlation Coefficient/*R*^2^
literature [84]	y=2.44 x + 1.07	0.972
literature [85]	y=2.904 x − 0.263	0.954
literature [86]	y=x + 8	0.97

**Table 7:** Correlation coefficients of the fitted model for the relationship between compressive strength of cementitious materials and fractal dimension $D_{max}$ in freeze–thaw cycles with regression parameter table.

Fit a Model (Math.)		Specimen 1	Specimen 2	Specimen 3
$Y={\mathrm{e}}^{ax-b}$	a	7.24	7.41	5.97
	b	18.17	18.67	4.41
	$R^{2}$	0.98	0.99	0.85

**Table 8:** Genomic information for cement-based materials.

	Grading Criteria			Characterized Genes	Associative Properties
cementitious material	Constituents	Nano	C-S-H	Calcium–silica ratio	Pore structure, early compressive strength, flow properties, frost resistance, stiffness
	Structural genes	Micro	Pore structure	Porosity, pore size distribution, pore shape	frost resistance, permeability, compressive strength
			Air content	Air content, bubble spacing factor	Frost resistance, durability, compression resistance
		Macro	Aggregate	Shape	Chloride penetration resistance, viscosity, fluidity
				Angularity	Fluidity, viscosity, strength, peak shear stress
				Surface texture	Slip resistance
				Crushing index	Compressive strength, flexural strength, modulus of elasticity, coefficient of permeability
				water absorption	Slump, fluidity, drying shrinkage capacity, compressive strength

## Data Availability

The raw data supporting the conclusions of this article will be made available by the authors on request.
